# Supplementary material for: SOX2 confers tumour permissiveness in a specific skin progenitor population
Source: Nat Commun. 2026 Jan 8;17:304. doi: 10.1038/s41467-025-66251-4 (PMC12789432; doi:10.1038/s41467-025-66251-4)
Supplement: Supplementary file 1 — Supplementary Information [file 41467_2025_66251_MOESM1_ESM.pdf]

Supplementary Information.

SOX2 confers tumour permissiveness in a specific skin progenitor population.

Patricia P. Centeno[1,2], Christopher Chester[2,†], Georgios Kanellos[1], Catriona A. Ford[1], Patrizia Cammareri[1, ‡], Gareth J. Inman[1,3], Thomas Jamieson[1], Rachel A Ridgway[1], Richard Marais[2,†], Andrew D. Campbell[1], Owen J. Sansom[1,3].

[1]Cancer Research UK Scotland Institute, Glasgow, UK.

[2]Cancer Research UK Manchester Institute, Manchester, UK.

[3]School of Cancer Sciences, University of Glasgow, UK.

[†]Present address: Oncodrug Ltd, Alderley Park, Macclesfield, UK.

[‡]Present address: Cancer Research UK Scotland Centre, Institute of Genetics and Cancer, University of Edinburgh, Edinburgh, UK.

Supplementary Fig. 1

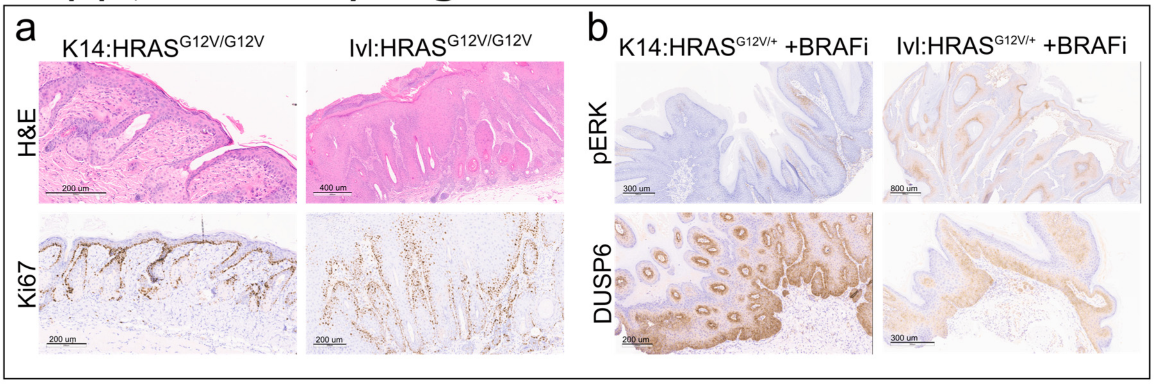

Supplementary Fig. 1. Histological characterisation of HRAS^{G12V} driven tumours, related to Figure 1.

a. Representative images of H&E (scale bars are 200 µm and 400 µm) and IHC of Ki67 (scale bar is 200 µm) conducted in skin derived from K14:HRAS$^{G12V/G12V}$ and tumours from Ivl:HRAS$^{G12V/G12V}$ at clinical endpoint. Images representative of four animals per genotype. **b**. Representative images of IHC of the downstream targets of MAPK signalling activation, pERK (scale bars are 300 µm and 800 µm) and DUSP6 (scale bars are 200 µm and 300 µm), conducted in tumours derived from K14:HRAS$^{G12V/+}$ and Ivl:HRAS$^{G12V/+}$ in combination with BRAFi at the clinical endpoint. Images representative of four animals per genotype.

Supplementary Fig. 2

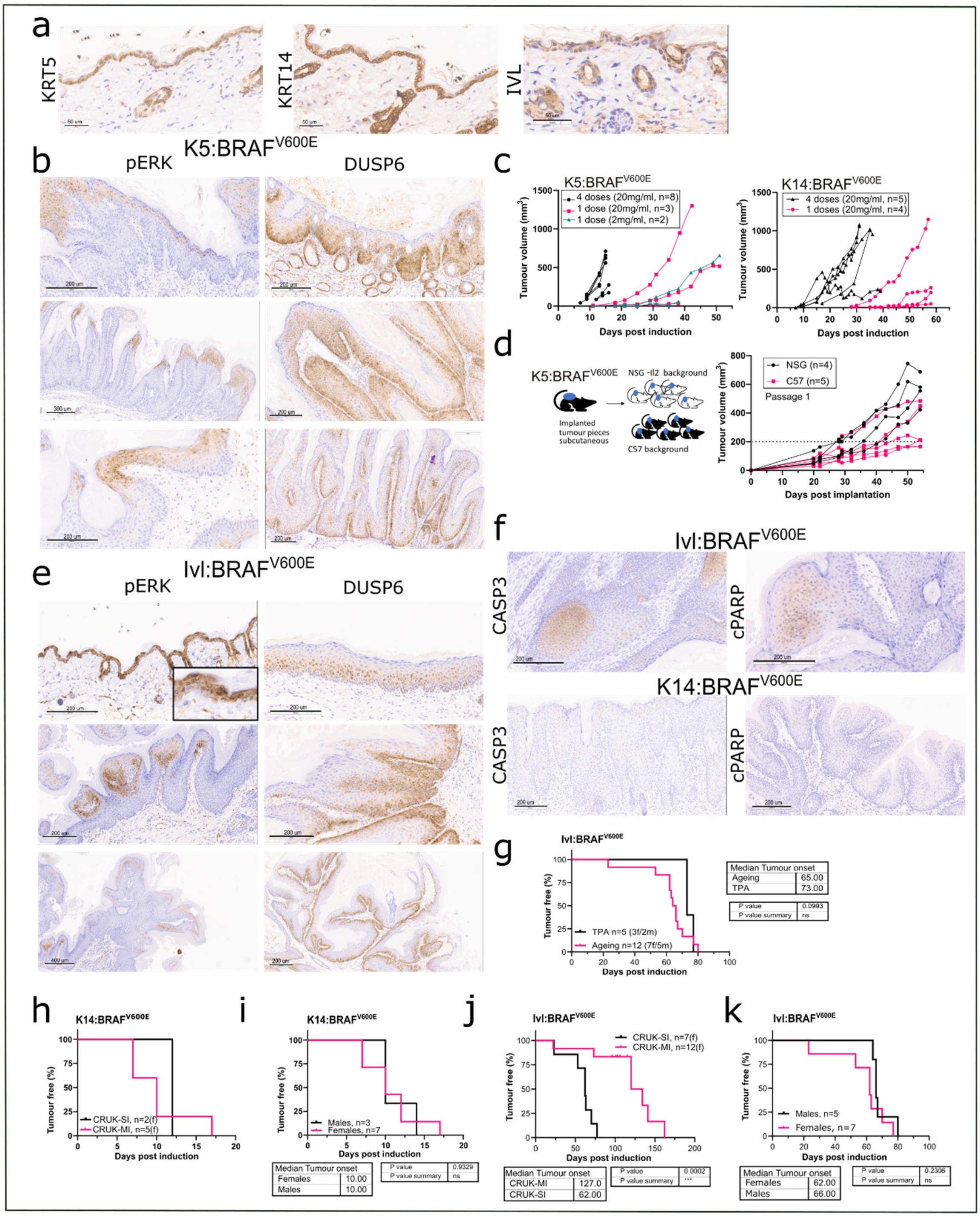

Supplementary Fig. 2. Tumour-primed and tumour-resistant populations coexist in the basal layer, related to Figure 2

a. Representative images of IHC staining of KRT5, KRT14 and IVL in normal wild-type skin. Images representative of five animals per genotype. Scale bar is 50 µm. **b**. Representative images of IHC of the downstream targets of MAPK signalling activation, pERK and DUSP6, conducted in tumours derived from K5:BRAF^{V600E} at clinical endpoint. Images representative of five animals per genotype. Scale bar is 200 µm unless otherwise stated in the image. **c**. Tumour burden growth at different induction regimens in K5:BRAF^{V600E} and K14:BRAF^{V600E} models. **d**. Schematic and tumour burden

growth of K5:BRAF^{V600E} tumour pieces transplanted into C57/6J (n=5) and immunosuppressed female mice (NSG-Il2; n=4). **e**. Representative images of IHC of the downstream targets of MAPK signalling activation, pERK and DUSP6, conducted in tumours derived from Ivl:BRAF^{V600E} at clinical endpoint. Images representative of five animals per genotype. Scale bar is 200 µm unless otherwise stated in the image. **f**. Representative images of IHC of the apoptotic markers cleaved CASP3 and cPARP, conducted in tumours derived from Ivl:BRAF^{V600E} and K14:BRAF^{V600E} at clinical endpoint. Images representative of five animals per genotype. Scale bar is 200 µm. **g**. Kaplan-Meier tumour-free survival plot for Ivl:BRAF^{V600E} female mice (n=12) aged until clinical endpoint and those treated with the tumour promoter TPA (n=5), conducted at the CRUK-Scotland Institute (CRUK-SI) facility. P-values were determined using the log-rank (Mantel-Cox) test. **h**. Kaplan-Meier tumour-free survival plots of female K14:BRAF^{V600E} mice, aged until clinical endpoint, housed at CRUK Manchester Institute (CRUK-MI; n=5) and CRUK-SI (n=2) facilities. Note that the CRUK-MI cohort is also shown in Figure 2c for comparison. **i**. Kaplan-Meier tumour-free survival plots of K14:BRAF^{V600E} male (n=3) and female (n=7). Note that the female cohort includes five mice housed at CRUK-MI, as shown in Figure 2c, and an additional two housed at CRUK-SI. All males were housed at CRUK-SI. P-values were determined using the log-rank (Mantel-Cox) test. **j.** Kaplan-Meier tumour-free survival plots of Ivl:BRAF^{V600E} female mice, aged until clinical endpoint at CRUK-SI (n=7) and CRUK-MI (n=12). Note that the CRUK-MI cohort is also shown in 2c for comparison. **k**. Kaplan-Meier tumour-free survival plots of Ivl:BRAF^{V600E} grouped by sex (males n=5 and females n=7). All mice were housed at CRUK-SI. P-values were determined using the log-rank (Mantel-Cox) test. Source data are provided as a Source Data file.

Supplementary Fig. 3

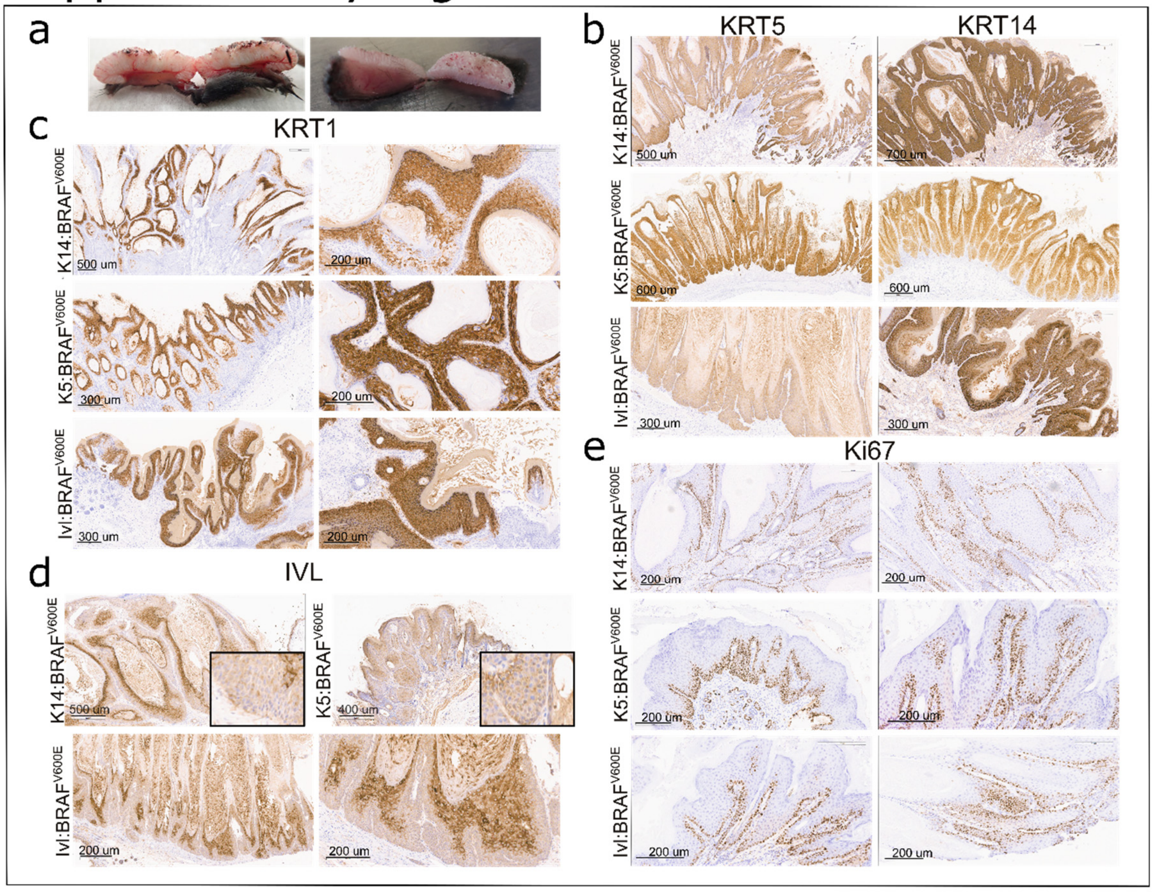

Supplementary Fig. 3. Histological features of BRAF^{V600E} driven tumours, related to Figure 2.

a. Representative macroscopic images of the tumours resulting from the K14/K5+ tumour-prime population showing demarcated borders and vertical columns of keratinocyte proliferation. Images representative of five animals per genotype. **b-e**. Representative IHC of skin hierarchy markers KRT5 and KRT14 (b), KRT1 (c), IVL (d), and proliferation marker Ki67 (e) from K14:BRAF^{V600E}, K5:BRAF^{V600E} and Ivl:BRAF^{V600E} tumours at clinical endpoint. Images representative of five animals per genotype. Scale bar is 200 µm unless otherwise stated in the image.

Supplementary Fig. 4

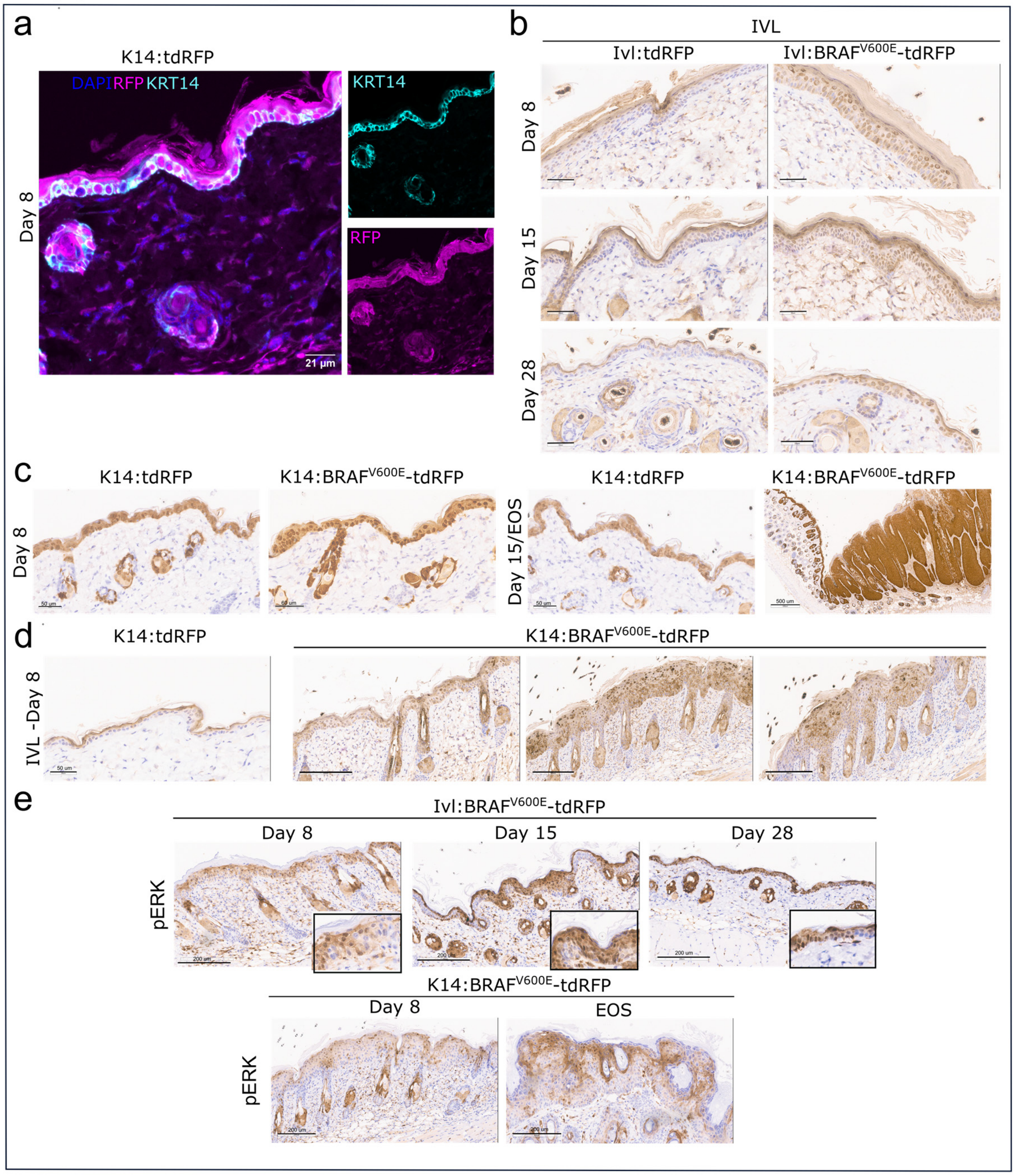

Supplementary Fig.4. Lineage tracing characterisation of Ivl and K14 models, related to Figure 3.

a. Representative Z-stack co-immunofluorescence (IF) of KRT14 and RFP of skin derived from K14:tdRFP (control) at day 8 post-induction. Images representative of three animals per genotype. Scale bar is 21 µm. **b**. Representative epidermal IHC of IVL at different time points post-induction in Ivl:tdRFP and Ivl:BRAF^{V600E}-tdRFP. Images representative of three animals per genotype. Scale bar is 50 µm. **c**. Representative IHC of RFP at different time points post-induction in K14:tdRFP and K14:BRAF^{V600E}-tdRFP epidermis, epidermis adjacent to tumour and tumour. Images representative of three animals per genotype. Scale bars are 50 µm, 60 µm and 500 µm. **d**. Representative IHC of IVL at different time points post-induction in K14:tdRFP (scale bar is 50 µm) and K14:BRAF^{V600E}-tdRFP epidermis, epidermis adjacent to tumour and tumour (scale bar is 200 µm). Images representative of three animals per genotype. **e**. Representative IHC of pERK at different time points post-induction in

Ivl:BRAF^{V600E}-tdRFP and K14:BRAF^{V600E}-tdRFP. Images representative of three animals per genotype. Scale bar is 200 µm.

Supplementary Fig. 5

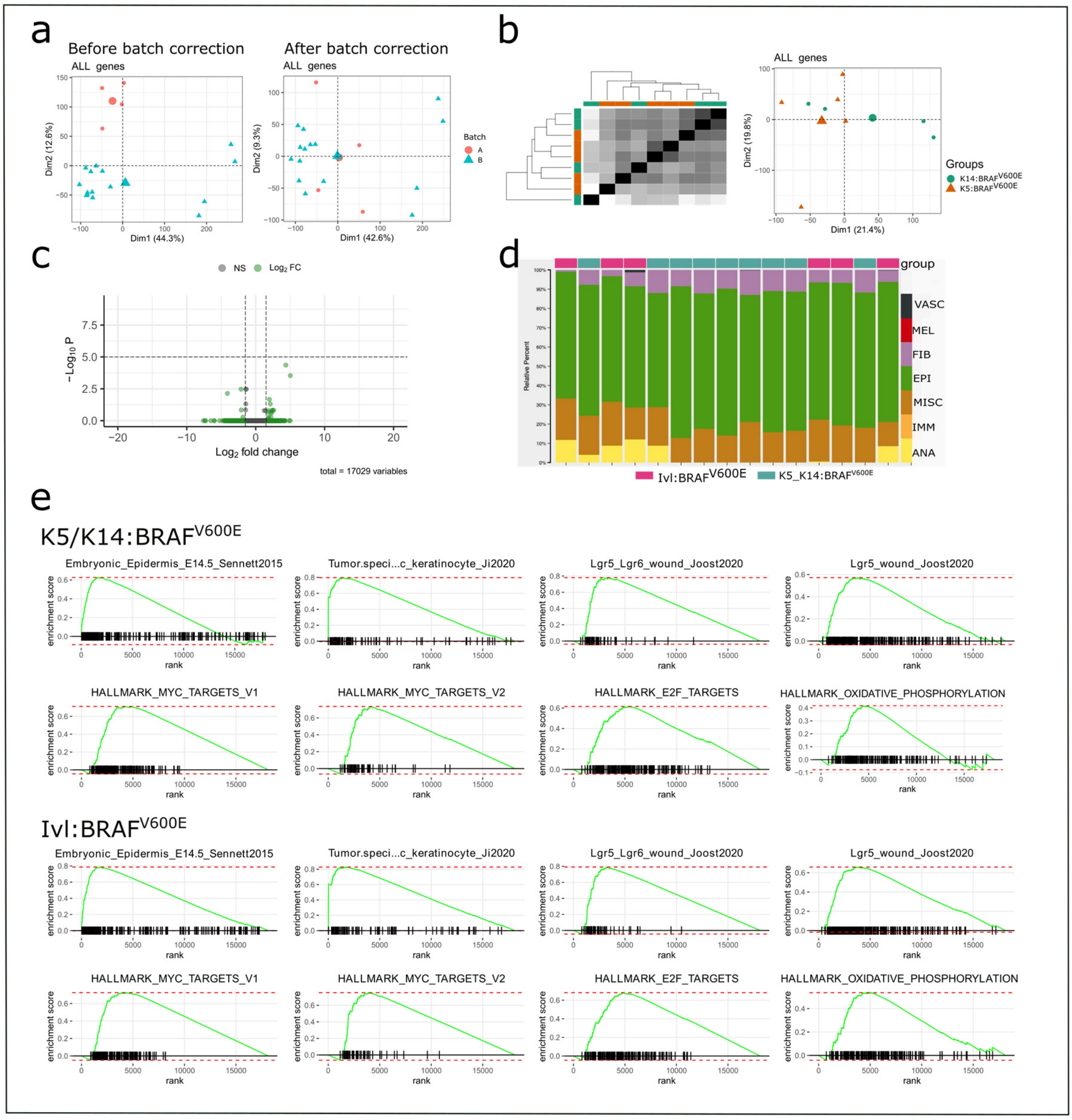

Supplementary Fig.5. Transcriptional landscape of tumour-primed K14/K5:BRAF^{V600E} and tumour-resistant Ivl:BRAF^{V600E}, related to Figure 4.

a. Principal component analysis (PCA) of normalised expression values before and after batch correction, showing the correlation between the two different batches sequenced at different times (batch A n=4 and batch B n=15). **b**. Hierarchical clustering heatmap and PCA of normalised expression values showing the correlation between the transcriptomes of the K5:BRAF^{V600E} (n=5) and K14:BRAF^{V600E} tumours (n=4). **c**. Volcano plot showing differentially expressed genes between K5:BRAF^{V600E} (n=5) and K14:BRAF^{V600E} (n=4) tumours using Wald test (two-tailed). NS, not significant. **d**. Digital sorting analysis of the bulk RNAseq transcriptome obtained from Ivl:BRAF^{V600E} (n=6) and K5/K14:BRAF^{V600E} (n=9) tumours, using CIBERSORTx and the single-cell RNAseq dataset from Joost and colleagues[1] for deconvolution including MEL (melanocytes), FIB (fibroblast), EPI (epidermis, permanent part), VASC (Vasculature), IMM (immune cells), ANA (anagen, hair follicle related keratinocytes), and MISC (miscellaneous, mixed cells or not assigned) skin cell populations. **e**. Selected gene set enriched Hallmarks and pathways in K5/K14:BRAF^{V600E} (n=9) and Ivl:BRAF^{V600E} (n=6)

tumours at clinical endpoint. Pathways shown are significantly enriched (padj <0.01 based on an adaptive multi-level split Monte-Carlo).

Supplementary Fig. 6

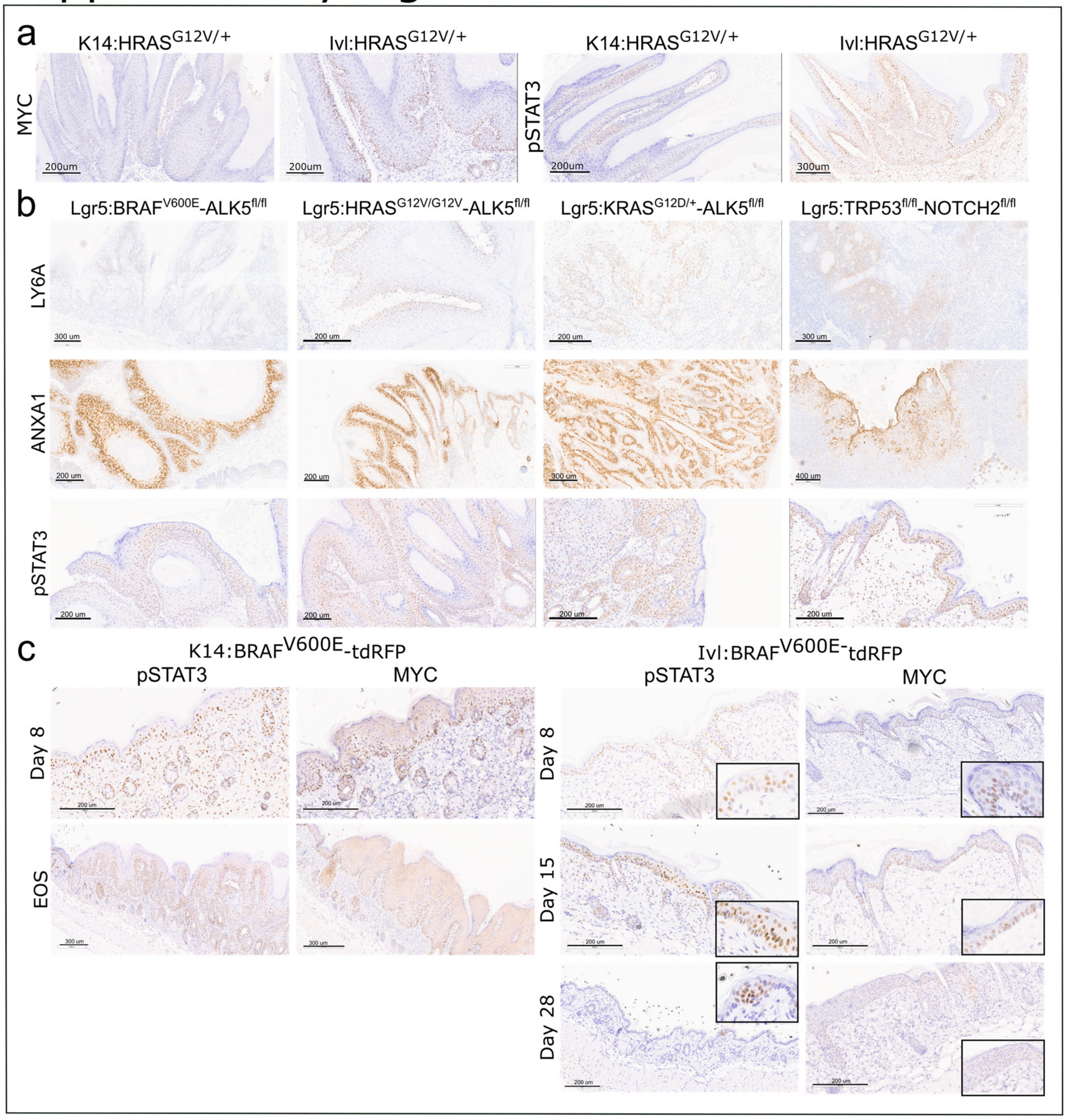

Supplementary Fig. 6. Shared transcriptional landscape, related to Figure 4.

a. Representative images of IHC validation of MYC and pSTAT3 in tumours derived from the K14:HRAS$^{G12V/+}$ and Ivl:HRAS$^{G12V/+}$ models treated with BRAFi. Scale bar is 200 µm unless otherwise stated in the image. Images representative of three animals per genotype. **b**. Representative images of IHC validation of selected targets in Lgr5:BRAF^{V600E}-ALK5$^{fl/fl}$, Lgr5:HRAS$^{G12V/G12V}$-ALK5$^{fl/fl}$, Lgr5:KRAS$^{G12D/+}$-ALK5$^{fl/fl}$ and Lgr5:TRP53$^{fl/fl}$-NOTCH2$^{fl/fl}$ tumours at clinical endpoint. Scale bar is 200 µm unless otherwise stated in the image. Images representative of three animals per genotype. **c**. Representative images of IHC validation of MYC and pSTAT3 in skin sampled at the indicated time points derived from the K14:BRAF^{V600E} and Ivl:BRAF^{V600E} models. Images representative of three animals per genotype. Scale bar is 200 µm unless otherwise stated in the image.

Supplementary Fig. 7

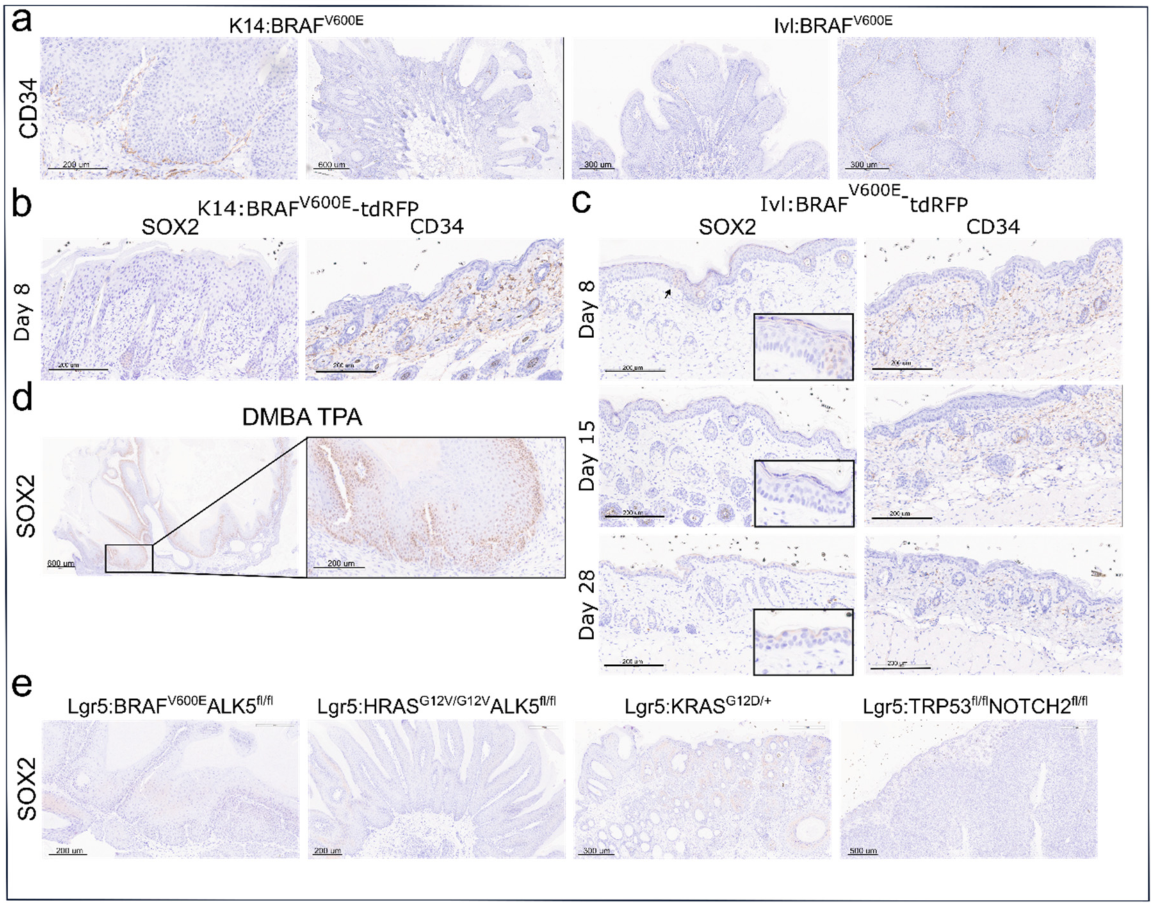

Supplementary Fig. 7. Characterisation of tumour-initiating cells marked by SOX2 and CD34 across models, related to Figure 5.

a. Representative IHC of CD34 in K14:BRAF^{V600E} and Ivl:BRAF^{V600E} derived tumours. Images representative of three animals per genotype. Scale bars are 200 µm, 300 µm and 600 µm. **b,c**. Representative IHC of CD34 and SOX2 in epidermis sampled at the indicated time points post-induction in K14:BRAF^{V600E}-tdRFP(b) and Ivl:BRAF^{V600E}-tdRFP epidermis (c). Images representative of three animals per genotype. Scale bar is 200 µm. **d,e**. Representative images of IHC of SOX2 in DMBA/TPA-derived tumours (d), and tumours derived from the *Lgr5* population with different driver combinations: Lgr5:BRAF^{V600E}-ALK5, Lgr5:HRAS^{G12V}-ALK5, Lgr5:KRAS^{G12D}-ALK5 and Lgr5:TRP53-NOTCH2 at clinical endpoint (e). Images representative of three animals per genotype. Scale bar is 200 µm unless otherwise stated in the image.

Supplementary Fig. 8

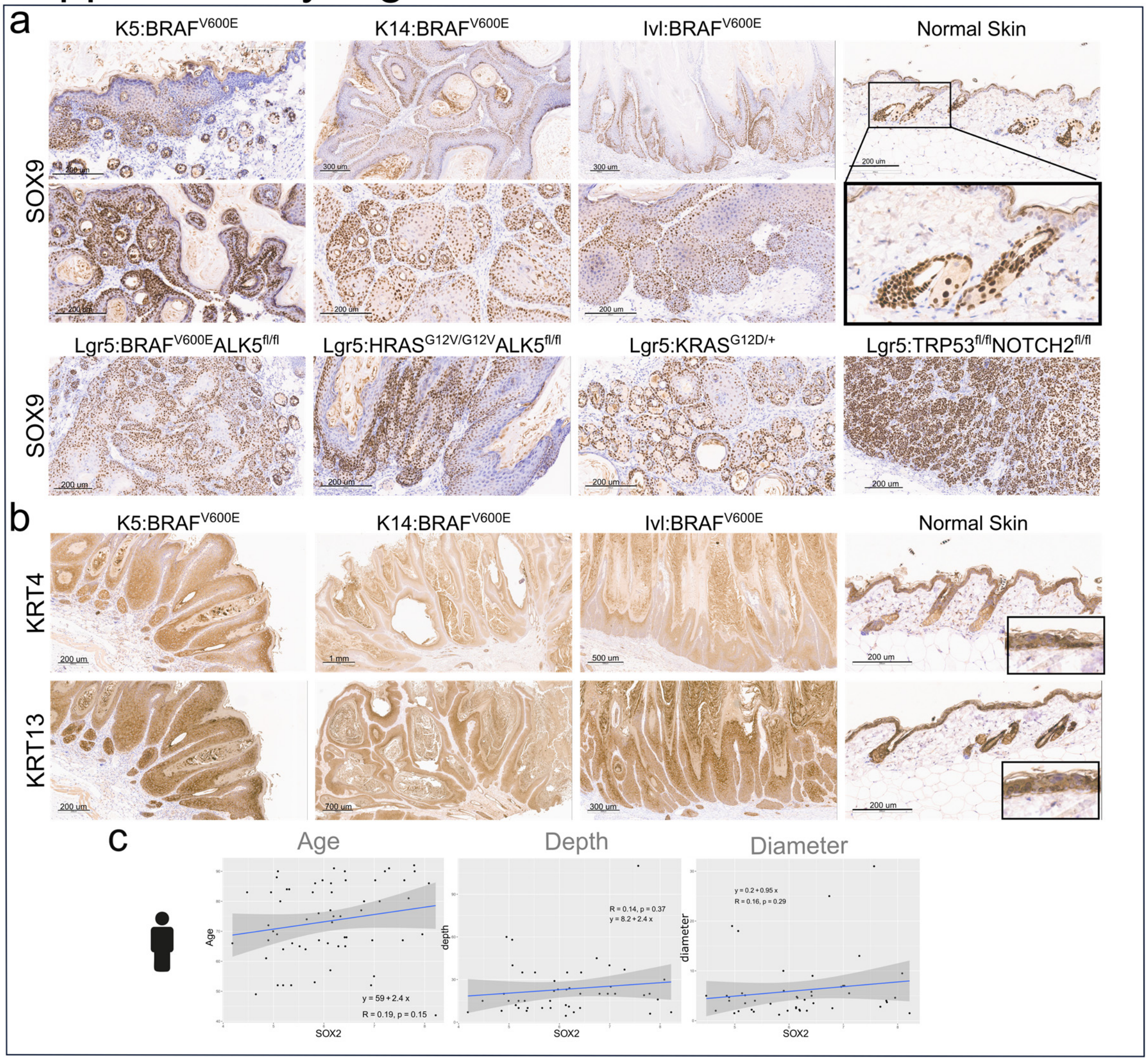

Supplementary Fig. 8. Characterisation of non-cell-specific markers, related to Figure 5.

a. Representative images of IHC of SOX9 in a range of tumours, including K5:BRAF^{V600E}, K14:BRAF^{V600E}, Ivl:BRAF^{V600E}, Lgr5:BRAF^{V600E}-ALK5$^{fl/fl}$, Lgr5:HRAS$^{G12V/G12V}$-ALK5$^{fl/fl}$, Lgr5:KRAS$^{G12D/+}$-ALK5$^{fl/fl}$ and Lgr5:TRP53$^{fl/fl}$-NOTCH2$^{fl/fl}$ at clinical endpoint, and normal skin showing predominant expression in the hair follicle cells. Images representative of three animals per genotype. Scale bar is 200 µm unless otherwise stated in the image. **b**. Representative images of IHC of KRT4 and KRT13 in K5:BRAF^{V600E}, K14:BRAF^{V600E} and Ivl:BRAF^{V600E} tumours and in normal skin showing expression throughout the epidermal layers. Images representative of three animals per genotype. Scale bars are 200 µm, 300 µm, 500 µm, 700 µm and 1 mm. **c**. Normalised SOX2 levels from the human cSCC dataset[2] show no correlation with age, depth or diameter.

Supplementary Fig. 9

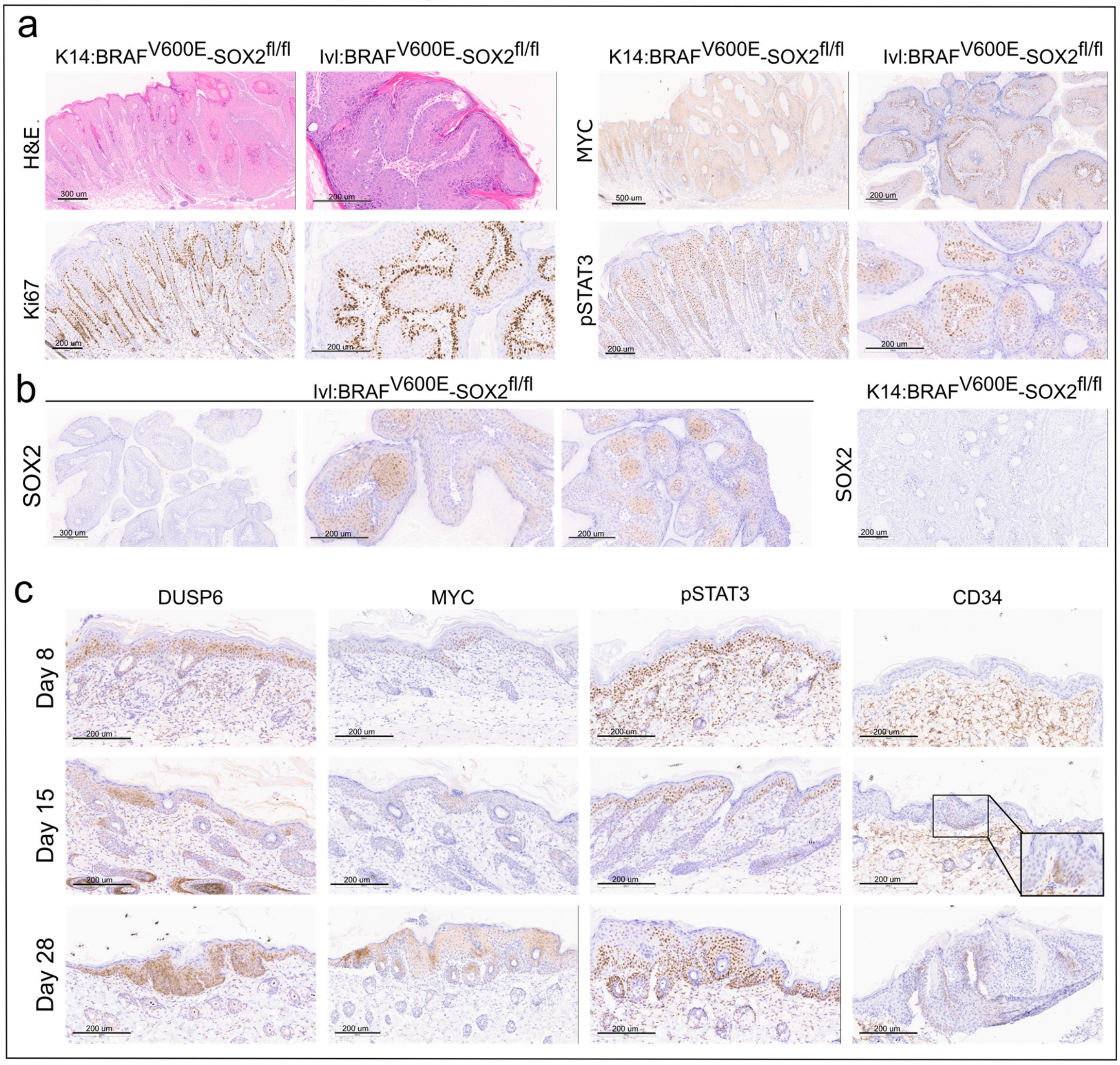

Supplementary Fig. 9. SOX2 renders the IVL+ tumour-resistant population susceptible to tumorigenesis and accelerates transformation, related to Figure 6.

a. Representative H&E and IHC of Ki67, MYC and pSTAT3 conducted in K14:BRAF^{V600E}-SOX2$^{fl/fl}$ and Ivl:BRAF^{V600E}-SOX2$^{fl/fl}$ tumours at clinical endpoint. Images representative of four animals per genotype. Scale bar is 200 µm unless otherwise stated in the image. **b**. IHC validation of SOX2 expression in Ivl:BRAF^{V600E}-SOX2$^{fl/fl}$ tumours, revealing partial reexpression of SOX2 and no expression in K14:BRAF^{V600E}-SOX2$^{fl/fl}$. Images representative of four animals per genotype. Scale bar is 200 µm unless otherwise stated in the image. **c**. Representative images of IHC of the downstream targets of MAPK signalling activation DUSP6, MYC, pSTAT3, and CD34, conducted in skin derived from Ivl:BRAF^{V600E}-SOX2$^{fl/fl}$ at the indicated time points. Images representative of three animals per genotype. Scale bar is 200 µm.

Supplementary Fig.10

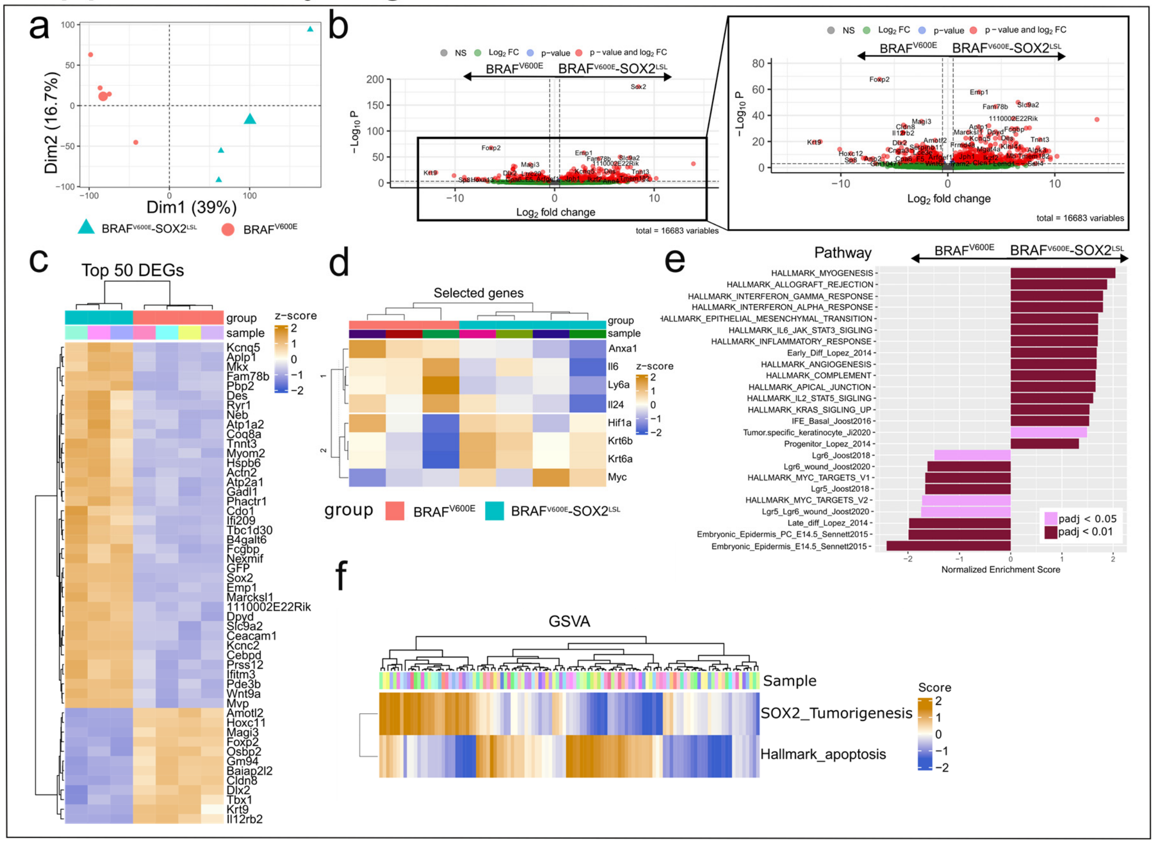

Supplementary Fig. 10. SOX2 accelerates the transformation from the IVL+ tumour-resistant population, related to Figure 6.

a. PCA of normalised expression showing the correlation between Ivl:BRAF^{V600E} (n=4) and Ivl:BRAF^{V600E}-SOX2^{LSL} (n=3) sequenced in one batch. **b**. Volcano plot showing differentially expressed genes between Ivl:BRAF^{V600E} (n=4) and Ivl:BRAF^{V600E}-SOX2^{LSL} (n=3) tumours using Wald test (two-tailed). **c,d**. Hierarchical clustering heatmap of top 50 differentially expressed genes (c) and selected genes (d) showing normalised expression and correlation between Ivl:BRAF^{V600E} (n=6) and Ivl:BRAF^{V600E}-SOX2^{LSL} (n=3). **e.** Gene set enrichment analysis of Hallmarks and indicated pathways in Ivl:BRAF^{V600E} (n=6) and Ivl:BRAF^{V600E}-SOX2^{LSL} (n=3). Showing pathways significantly enriched (at least padj <0.05, based on an adaptive multi-level split Monte-Carlo). **f**. Single-sample Gene Set Variation Analysis (GSVA) for selected signatures in cSCC samples obtained from a human dataset (n=62) [2].

Supplementary reference list

1. Joost, S. *et al.* The Molecular Anatomy of Mouse Skin during Hair Growth and Rest. *Cell Stem Cell* **26**, 441-457.e7 (2020).

2. Bailey, P. *et al.* Driver gene combinations dictate cutaneous squamous cell carcinoma disease continuum progression. *Nat Commun* **14**, 5211 (2023).
