# Supplementary material for: SOX2 confers tumour permissiveness in a specific skin progenitor population
Source: Nat Commun. 2026 Jan 8;17:304. doi: 10.1038/s41467-025-66251-4 (PMC12789432; doi:10.1038/s41467-025-66251-4)
Supplement: Supplementary file 2 — Description of Additional Supplementary Information [file 41467_2025_66251_MOESM2_ESM.pdf]

**Description of Additional Supplementary Information**

Supplementary Data 1. cSCC MAPK signalling shared signature derived from commonly upregulated and differentially expressed genes ($Log_2$ Fold Change >3 and padj <0.01) obtained from Ivl:BRAF$^{V600E}$ , K5:BRAF$^{V600E}$ and K14:BRAF$^{V600E}$ tumours compared to normal skin from control litter mates.

Supplementary Data 2. SOX2_tumorigenesis_human signature derived from the top 150 differentially expressed genes ($Log_2$ Fold Change >2 and padj <0.01) between Ivl:BRAF$^{V600E}$ and Ivl:BRAF$^{V600E}$-SOX2$^{LSL}$ murine tumours of which 139 genes were converted to human orthologs.
