# Supplementary material for: SOX2 confers tumour permissiveness in a specific skin progenitor population
Source: Nat Commun. 2026 Jan 8;17:304. doi: 10.1038/s41467-025-66251-4 (PMC12789432; doi:10.1038/s41467-025-66251-4)
Supplement: Supplementary file 5 — Reporting Summary [file 41467_2025_66251_MOESM5_ESM.pdf]

nature portfolio

Corresponding author(s): Owen J Sansom
Patricia P Centeno

Last updated by author(s): 20th October 2025

Reporting Summary

Nature Portfolio wishes to improve the reproducibility of the work that we publish. This form provides structure for consistency and transparency in reporting. For further information on Nature Portfolio policies, see our Editorial Policies and the Editorial Policy Checklist.

Statistics

For all statistical analyses, confirm that the following items are present in the figure legend, table legend, main text, or Methods section.

n/a	Confirmed	
☐	☒	The exact sample size (*n*) for each experimental group/condition, given as a discrete number and unit of measurement
☐	☒	A statement on whether measurements were taken from distinct samples or whether the same sample was measured repeatedly
☐	☒	The statistical test(s) used AND whether they are one- or two-sided<br>*Only common tests should be described solely by name; describe more complex techniques in the Methods section.*
☐	☒	A description of all covariates tested
☐	☒	A description of any assumptions or corrections, such as tests of normality and adjustment for multiple comparisons
☐	☒	A full description of the statistical parameters including central tendency (e.g. means) or other basic estimates (e.g. regression coefficient) AND variation (e.g. standard deviation) or associated estimates of uncertainty (e.g. confidence intervals)
☐	☒	For null hypothesis testing, the test statistic (e.g. *F*, *t*, *r*) with confidence intervals, effect sizes, degrees of freedom and *P* value noted<br>*Give P values as exact values whenever suitable.*
☒	☐	For Bayesian analysis, information on the choice of priors and Markov chain Monte Carlo settings
☐	☒	For hierarchical and complex designs, identification of the appropriate level for tests and full reporting of outcomes
☐	☒	Estimates of effect sizes (e.g. Cohen's *d*, Pearson's *r*), indicating how they were calculated

Our web collection on statistics for biologists contains articles on many of the points above.

Software and code

Policy information about availability of computer code

Data collection	*Provide a description of all commercial, open source and custom code used to collect the data in this study, specifying the version used OR state that no software was used.*
Data analysis	For mouse samples raw sequence quality control was performed using FastQC (v0.11.8) before and after removing adapters and low-quality base calls (Phred score <20) using TrimGalore with default options (v0.6.6). Trimmed reads were aligned to GRCm38, release 100 using Hisat2 (v2.1.0). Gene counts were subsequently estimated using featurecounts (subread/1.6.3). For human cell lines, raw sequence quality control and alignment were performed using Nextfow nf-core/rnaseq pipeline using STAR and Salmon 52. Trimmed reads were aligned to GRCh38, release 113. Sva::ComBat-seq was used for batch correction (v3.52.0). For all samples, after removing transcripts without a minimum of 5 reads in at least one sample, the differential expression analysis between mouse tumours and skin was performed using the R package DESeq2 (v1.44.0). The resultant p-values were corrected for multiple comparisons using the Benjamini-Hochberg approach. The following additional R packages were used for downstream analysis: pheatmap (v1.0.8), fgsea (v1.30.0), enhancedVolcano (v1.22.0), clusterProfiler (v4.12.6), dplyr (v1.1.4), tidyr (v1.3.1), AnnotationHub (v3.12.0), ggvenn (0.1.10), AnnotationDbi (v1.66.0), mixOmics (v6.26.0).

For manuscripts utilizing custom algorithms or software that are central to the research but not yet described in published literature, software must be made available to editors and reviewers. We strongly encourage code deposition in a community repository (e.g. GitHub). See the Nature Portfolio guidelines for submitting code & software for further information.

Data

Policy information about availability of data

All manuscripts must include a data availability statement. This statement should provide the following information, where applicable:

- Accession codes, unique identifiers, or web links for publicly available datasets
- A description of any restrictions on data availability
- For clinical datasets or third party data, please ensure that the statement adheres to our policy

The data discussed in this publication have been deposited in NCBI's Gene Expression Omnibus. The accession numbers for the RNA-seq data reported in this paper are NCBI GEO GSE280236 and GSE303703. Source Data are provided with this paper. The remaining data are available within the Article, Supplementary Information or Source Data file.

Research involving human participants, their data, or biological material

Policy information about studies with human participants or human data. See also policy information about sex, gender (identity/presentation), and sexual orientation and race, ethnicity and racism.

Reporting on sex and gender	*Use the terms sex (biological attribute) and gender (shaped by social and cultural circumstances) carefully in order to avoid confusing both terms. Indicate if findings apply to only one sex or gender; describe whether sex and gender were considered in study design; whether sex and/or gender was determined based on self-reporting or assigned and methods used. Provide in the source data disaggregated sex and gender data, where this information has been collected, and if consent has been obtained for sharing of individual-level data; provide overall numbers in this Reporting Summary. Please state if this information has not been collected. Report sex- and gender-based analyses where performed, justify reasons for lack of sex- and gender-based analysis.*
Reporting on race, ethnicity, or other socially relevant groupings	*Please specify the socially constructed or socially relevant categorization variable(s) used in your manuscript and explain why they were used. Please note that such variables should not be used as proxies for other socially constructed/relevant variables (for example, race or ethnicity should not be used as a proxy for socioeconomic status). Provide clear definitions of the relevant terms used, how they were provided (by the participants/respondents, the researchers, or third parties), and the method(s) used to classify people into the different categories (e.g. self-report, census or administrative data, social media data, etc.) Please provide details about how you controlled for confounding variables in your analyses.*
Population characteristics	*Describe the covariate-relevant population characteristics of the human research participants (e.g. age, genotypic information, past and current diagnosis and treatment categories). If you filled out the behavioural & social sciences study design questions and have nothing to add here, write "See above."*
Recruitment	*Describe how participants were recruited. Outline any potential self-selection bias or other biases that may be present and how these are likely to impact results.*
Ethics oversight	*Identify the organization(s) that approved the study protocol.*

Note that full information on the approval of the study protocol must also be provided in the manuscript.

Field-specific reporting

Please select the one below that is the best fit for your research. If you are not sure, read the appropriate sections before making your selection.

☒ Life sciences ☐ Behavioural & social sciences ☐ Ecological, evolutionary & environmental sciences

For a reference copy of the document with all sections, see nature.com/documents/nr-reporting-summary-flat.pdf

Life sciences study design

All studies must disclose on these points even when the disclosure is negative.

Sample size	A priori based on historical data sets were used to ensure the smallest sample size that could give a significant difference was chosen in accordance with the 3Rs
Data exclusions	No data were excluded.
Replication	Multiple biological replicates were used to verify reproducibility of experiments. All attempts at replication were successful.
Randomization	For all histological analysis the samples were randomized
Blinding	For all histological analysis the samples researchers were blinded to the genotype or treatment

Reporting for specific materials, systems and methods

We require information from authors about some types of materials, experimental systems and methods used in many studies. Here, indicate whether each material, system or method listed is relevant to your study. If you are not sure if a list item applies to your research, read the appropriate section before selecting a response.

Materials & experimental systems

n/a	Involved in the study
☐	☒ Antibodies
☒	☐ Eukaryotic cell lines
☒	☐ Palaeontology and archaeology
☐	☒ Animals and other organisms
☒	☐ Clinical data
☒	☐ Dual use research of concern
☒	☐ Plants

Methods

n/a	Involved in the study
☒	☐ ChIP-seq
☒	☐ Flow cytometry
☒	☐ MRI-based neuroimaging

Antibodies

Antibodies use	KRT5 (905501, Biolegend), cleaved CASP3 (9661, Cell Signalling), cPARP (ab32064, Abcam),CD34 (119302, Biolegend), Ki67 (12202, Cell Signaling), SOX2 (14962, Cell Signaling) and pSTAT3 (9131, Cell Signaling). anti-rabbit EnVision HRP-conjugated secondary antibody (K4003, Agilent). KRT14 (ab7800, Abcam), c-MYC (ab32072, Abcam), RFP (600-401-379, Rockland) and SOX9 (AB5535, Millipore). In-situ hybridisation detection for Anxa1 (509298), Ivl (422538), Ly6a (427578), PPIB (313918; positive control) and dapB (312038; negative control) (all Bio-Techne) mRNA. was performed using RNAScope 2.5 LSx (Brown) detection kit (322700; Bio-Techne) according to the manufacturer's instructions. H&E staining was performed on a Leica autostainer (ST5020). Goat anti-rabbit IgG 488 (A11034, Invitrogen) and goat anti-mouse IgG 647 secondary antibody (A21236, Invitrogen diluted), DAPI (MBD0015, Sigma-Aldrich). Primary antibody application optimal dilution:KRT5, 1/1500; Ki67, 1/1000; pSTAT3, 1/100; SOX2, 1/200; CASP3, 1/500. KRT14, 1/300; c-MYC, 1/800; RFP, 1/1000; SOX9, 1/500; CD34, 1/100; cPARP 1/1000
Validation	All antibodies were selected on the manufacturers recommendations regarding target species. Antibodies were optimised and validated on control tissue with known expression of target antigen; location and intensity of signal where assessed to confirm correct antibody binding.

Eukaryotic cell lines

Policy information about cell lines and Sex and Gender in Research

Cell line source(s)	*State the source of each cell line used and the sex of all primary cell lines and cells derived from human participants or vertebrate models.*
Authentication	*Describe the authentication procedures for each cell line used OR declare that none of the cell lines used were authenticated.*
Mycoplasma contamination	*Confirm that all cell lines tested negative for mycoplasma contamination OR describe the results of the testing for mycoplasma contamination OR declare that the cell lines were not tested for mycoplasma contamination.*
Commonly misidentified lines (See ICLAC register)	*Name any commonly misidentified cell lines used in the study and provide a rationale for their use.*

Animals and other research organisms

Policy information about studies involving animals; ARRIVE guidelines recommended for reporting animal research, and Sex and Gender in Research

Laboratory animals	Mus Musculus with a mixed C57BL/6 background were used, experiments were started on mice aged 6-10 weeks.
Wild animals	Study did not involve wild animals
Reporting on sex	Mouse sex was determined at weaning by inspecting the genitalia. Numbers of males and females used per experimental group is reported for each experiment in the figure and figure legends. Sex differences were not seen.
Field-collected samples	Study did not involve samples collected from the field
Ethics oversight	All mouse experiments were performed according to UK Home Office regulations (project licence 70/8646 and PP3908577) following approval by the University of Glasgow Animal Welfare and Ethical Review Body.

Note that full information on the approval of the study protocol must also be provided in the manuscript.

Plants

Seed stocks	*Report on the source of all seed stocks or other plant material used. If applicable, state the seed stock centre and catalogue number. If plant specimens were collected from the field, describe the collection location, date and sampling procedures.*
Novel plant genotypes	*Describe the methods by which all novel plant genotypes were produced. This includes those generated by transgenic approaches, gene editing, chemical/radiation-based mutagenesis and hybridization. For transgenic lines, describe the transformation method, the number of independent lines analyzed and the generation upon which experiments were performed. For gene-edited lines, describe the editor used, the endogenous sequence targeted for editing, the targeting guide RNA sequence (if applicable) and how the editor was applied.*
Authentication	*Describe any authentication procedures for each seed stock used or novel genotype generated. Describe any experiments used to assess the effect of a mutation and, where applicable, how potential secondary effects (e.g. second site T-DNA insertions, mosiacism, off-target gene editing) were examined.*
